# Ultra‐Processed Foods and Markers of Systemic Inflammation in Children

**DOI:** 10.1002/fsn3.70795

**Published:** 2025-09-01

**Authors:** Camila Awad, Paola Rubilar, Macarena Hirmas‐Adauy, Verónica Iglesias, María Pía Muñoz, Mauricio A. Retamal, Cristóbal Carvajal, Payam Dadvand, Camille Lassale

**Affiliations:** ^1^ Universitat Pompeu Fabra Barcelona Spain; ^2^ ISGlobal Barcelona Spain; ^3^ Centro de Epidemiología y Políticas de Salud, Facultad de Medicina Clínica Alemana Universidad del Desarrollo Santiago Chile; ^4^ Escuela de Salud Pública, Facultad de Medicina Universidad de Chile Santiago Chile; ^5^ Centro para la Prevención y el Control del Cáncer (CECAN) Santiago Chile; ^6^ Programa de Doctorado en Salud Pública, Facultad de Medicina Universidad de Chile Santiago Chile; ^7^ Programa de Comunicación Celular en Cáncer, Instituto de Ciencias e Innovación en Medicina, Facultad de Medicina Clínica Alemana Universidad del Desarrollo Santiago Chile; ^8^ Centro de Informática Biomédica, Facultad de Medicina, Clínica Alemana Universidad del Desarrollo Santiago Chile; ^9^ CIBER Epidemiología y Salud Pública (CIBERESP) Madrid Spain; ^10^ CIBER Physiopathology of Obesity and Nutrition (CIBEROBN) Madrid Spain

**Keywords:** biomarkers, dietary, inflammation, NOVA, primary‐school, ultra‐processed food

## Abstract

Diets high in ultra‐processed foods (UPF) have been associated with negative health outcomes in adults; however, UPF's impact on children's health and their underlying mechanisms remain underexplored, despite the rising prevalence of their intake in younger populations. We aimed to investigate the association between UPF intake and systemic inflammation in primary school children. This study included 450 children aged 7–10 years participating in a birth cohort in Arica, Chile (2023). Using the NOVA food classification system, we calculated the intake of UPF (expressed in %kcal) from a 44‐item food frequency questionnaire (FFQ). The associations between UPF intake (exposure) and an array of cytokine levels (IL‐6, IL‐8, IL‐10, IL‐12, IL1β, TNF‐α) were assessed using multiple linear regression models, adjusted for relevant covariates. The mean (SD) UPF intake was 29% (10.5%) of the total energy intake. Some of the associations appeared non‐linear; therefore, participants were grouped into tertiles of UPF intake. In adjusted models, a suggestive trend across tertiles was observed for IL‐1β (trend *p*‐value 0.01). Stratified analyses by age suggested an association between UPF intake and IL‐6 in older children (≥ 9 years) only (*p*‐value for interaction = 0.02). We found potential associations between UPF intake and cytokine levels in school‐aged children. These results may suggest inflammation as a mechanism underlying the adverse health consequences of UPF consumption in children.

## Graphical abstract

Illustration showing the association between ultra‐processed foods intake and systemic inflammation, measured with cytokines levels in children.

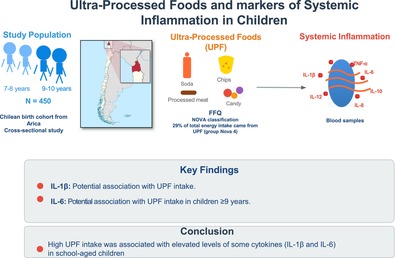

**Abbreviations**
BMI: body mass index
FFQ: Food Frequency Questionnaire
IL: interleukin
LLD: lower limit of detection
NCD: non‐communicable disease
TNF‐α: tumor necrosis factor α
UPF: ultra‐processed foods

## 1 Introduction

Ultra‐processed foods (UPF) are ready‐to‐eat formulations of processed substances that have been extracted or refined from whole foods. They usually contain added flavors, colors, and other additives, to enhance taste and appearance, but often provides limited nutritional value (Monteiro, Cannon, Levy, et al. 2019). Typically, these products have more than five ingredients, including substances with little or no culinary use and cosmetic additives‐some with unfamiliar or unrecognizable names (dos Santos Leffa 2023). Produced with industrial ingredients that alter the food's natural composition. UPF are engineered for profitability, as their production costs are far lower than the revenue they generate. (Lauria et al. 2021). Over the past decades, the consumption of UPF has surged (Monteiro et al. 2012), largely due to a dietary shift towards “Western diets” (Stolfi et al. 2023). In adults, in the United States (USA) and the United Kingdom (UK), nearly 60% of the total caloric intake comes from UPF (Grinshpan et al. 2023; Chavez‐Ugalde et al. 2024), while in Canada and Chile, this proportion is around 30%, 21% in Brazil (Cediel et al. 2018) and between 15% and 20% in European countries such as Spain, Sweden, or the Netherlands (Neri et al. 2022; Touvier et al. 2023). UPF are also being introduced at earlier ages, and it has been estimated that up to 60% of the total caloric intake of children comes from UPF, although this varies a lot across countries and socio‐demographic categories (Neri et al. 2022). A study on 545 infants, aged 24 months in Brazil showed that 74% of them consumed a type of UPF daily, such as candies, cookies, processed yogurts, soft drinks, or juices (Lopes et al. 2020). Two studies including primary school children in Europe and Chile found that 50% of the children's diet came from UPF (Lauria et al. 2021; Araya et al. 2021). UPF intake has been associated with a higher risk of obesity, type 2 diabetes (T2D), hypertension (Silva et al. 2023) cardiovascular disease, common mental disorders (depression, anxiety) (Lane et al. 2024) and cognitive disorders (Toloni et al. 2011) in adults. Gut microbiome alterations (Lane et al. 2022) and immune system impairments (Mescoloto et al. 2023) have been suggested as potential mechanisms underlying these associations. In children, an emerging body of evidence has associated UPF intake with altered lipid profiles, higher body mass index (BMI) and higher blood pressure; however, there has been heterogeneity in the reported associations (Rauber et al. 2015; Vilela et al. 2022; Bawaked et al. 2020).

Inflammation might be one of the underlying mechanisms behind the observed associations between UPF and deleterious health outcomes; a study also noted a negative association between UPF consumption and serum levels of Fibroblast Growth Factor‐19 (FGF‐19), which is involved in regulating bile acid synthesis and glucose metabolism, and its reduced levels may indicate metabolic disturbances related to inflammation (Xia et al. 2025). A diet high in UPF may induce endotoxemia, causing a persistent activation of the immune system, resulting in the proliferation of inflammatory mediators (Cani et al. 2007).

Cytokines are mediators of immune and inflammatory responses through a complex networking system. There is a vast range of cytokines such as interleukins (IL), TNF, interferons (IFN), lymphokines, monokines, and transforming growth factors (TGF), which are either classified as anti‐ or proinflammatory (Liu et al. 2021). Proinflammatory cytokines (e.g., TNF‐α, IL1β IL‐6, IL‐8, IL‐12, IFNγ) play a role in triggering and sustaining inflammation, while anti‐inflammatory cytokines (e.g., IL‐4, IL‐10, TGFβ, IL‐13) help reduce production of proinflammatory cytokines, limit tissue damage, and promote recovery during the acute stages of autoimmune diseases, among other actions (Moudgil and Choubey 2011). However, the categorization as pro‐ or anti‐inflammatory cytokine might be context dependent. For example, high levels of IL‐10 do not necessarily indicate an anti‐inflammatory state, as it may be elevated in response to counterbalance the effects of inflammatory cytokines (Doughty et al. 1998). IL‐10 may act as a compensatory mechanism to mitigate the harmful impact of excessive inflammation, rather than directly promoting immune regulation (de Vries 1995; Carlini et al. 2023). Elevated cytokine levels in children may indicate inflammatory states that are the hallmark of metabolic disturbances, immune health disorders, neurodevelopment disorders, obesity, risk of insulin resistance, and related inflammatory comorbidities, such as high blood pressure, altered lipid and glycemic profiles, among others (Bastos et al. 2024; Machado et al. 2019).

The emerging research on the impact of UPF intake on health outcomes, including cardiometabolic health in children (Vilela et al. 2022) underscores the need to better understand the underlying mechanisms of these associations. Although inflammation can potentially be one of these mechanisms, the available evidence on the effect of UPF intake on inflammation in children is very scarce. This study aimed to assess the relationship between UPF intake and inflammatory markers in primary school children.

## 2 Methods

### 2.1 Study Population

This study was nested in a birth cohort including 1644 pregnant women recruited between 2013 and 2016 by the Health Authority of the Region of Arica y Parinacota (Rubilar et al. 2025). They attended Hospital Juan Noé, the main hospital of the region of Arica and Parinacota, Chile. In 2023, the mothers were re‐contacted to conduct the follow‐up study of their children. Of all mothers included at baseline, 980 (58.5%) were located while the other 42.5% of the mothers could not be reached, since their contacts were out of date or did not respond to the invitations to participate. From the located mothers, 782 agreed to participate in the follow‐up, representing 46.7% of the initial cohort and 79.8% of those contacted. The main reasons for not participating were relocation to a different city or lack of interest in participating. From those 782 mothers, we randomly selected 443, including a total of 450 children aged 7–10 years (seven sibling pairs among them) who were included in our study. Informed consent was obtained from the parents/carers (thereafter termed “carer”) while children gave their verbal assent. This study was approved by the Scientific Ethics Committee of the University of Desarrollo, Santiago, Chile (Approval number: 2022.81).

### 2.2 Ultra‐Processed Food Assessment

The NOVA classification system (Monteiro, Cannon, Lawrence, et al. 2019) classifies foods into four groups based on their level of processing. Group 1 includes unprocessed or minimally processed foods such as oats or plain yogurt. Group 2 consists of processed culinary ingredients, such as oils and butter, used for cooking. Group 3 comprises processed foods with added salt, sugar, or preservatives, such as canned vegetables and cured meats. Group 4 (UPF) includes items such as sausages, margarine, refined cereals, industrial pastries, cookies, snacks, carbonated and sugary drinks, and additives.

A previously validated Food Frequency Questionnaire (FFQ) (Goni Mateos et al. 2016) was used, which asked about the child's diet over the last 12 months and the frequency of consumption. The frequency was categorized as never, once a month, two to three times a month, one to two times a week, three to four times a week, five to six times a week, once daily, two to three times daily, four to five times daily, and more than six times daily. The FFQ included 44 food items, encompassing local foods (Table S1). From the FFQ, seven were classified as UPF (NOVA group 4): high fat red meat (hamburgers and sausages), margarine, cereals, pastries, sugar, cooked or baked goods, and sugary drinks. In 86.4% of cases, the survey was answered by the mother, 6.4% by the father, and 7.1% by another carer. The quantity of UPF was calculated according to the Chemical Composition of Chilean Food Items (Schmidt Hebbel et al., n.d.), quantifying the grams and energy (kilocalories) of each food item. The intake of UPF was then expressed as the proportion (%) of the total energy intake of each participant (Araya et al. 2021; Machado et al. 2019).

### 2.3 Systemic Inflammation Assessment

Following the completion of the survey, a blood sample (4 mL) was obtained by a trained nurse through venipuncture using yellow‐top vacutainer tubes (containing a gel separator and thrombin). After centrifugation, serum samples were frozen at −80°C and stored at the University of Tarapacá in Arica. They were shipped to the Genetics Laboratory at the Faculty of Medicine, Universidad del Desarrollo, in Santiago, Chile, for analysis in November 2023. Cytokine levels were analyzed using flow cytometry on a CytoFLEX LX cytometer (Beckman Coulter, Brea, CA, USA). The BD Multiplex Cytometric Bead Array (CBA) protocol was followed to simultaneously measure multiple cytokines in samples: IL‐1β, IL‐6, IL‐8, IL‐10, IL‐12, and TNF‐α. According to the manufacturer, the lower limits of detection for IL1β, IL‐6, IL‐8, IL‐10, IL‐12p70, and TNF‐α were 7.2 g/mL, 2.6 pg/mL, 3.6 pg/mL, 3.3 pg/mL, 1.9 pg/mL, and 3.7 pg/mL, respectively.

### 2.4 Covariates

Children's age, sex, chronic disease diagnosis, indigenous group, carer's education, and healthcare affiliation were considered as potential confounders. Chronic disease diagnosis of the child encompassed clinically diagnosed diseases including cancer, asthma, allergic rhinitis, food allergy, insulin resistance, diabetes mellitus, obesity, dyslipidemia, hypertension, human immunodeficiency virus (HIV) infection, and kidney diseases. Carer's level of education was categorized into two groups: < 12 years of education and ≥ 12 years. Belonging to an indigenous group (i.e., Mapuche, Diaguita, Aymara, Rapanui, Colla, Quechua, Kawesqar, Yagan, or Chango) was categorized as Yes or No, according to the indigenous groups present in Chile. Regarding the healthcare affiliation, it was categorized as public or private/armed forces. Environmental tobacco smoke exposure was not included in the main statistical model because the large majority (82%) of the surveyed children had not been exposed to environmental tobacco smoke in the last 72 h before the survey. Body mass index (BMI, weight [kg]/height [m^2^]) *z*‐scores were calculated based on the World Health Organization's (WHO) 2007 growth standards (Growth Charts 2024). The weight status categories were defined according to BMI *z*‐score as underweight (≤ −1), normal weight (−1 to < 1), overweight (+1 to < 2), and obese (≥ 2) (WHO 2006); however, this variable was not included as a covariate in the main analysis, since it could be considered a mediator between UPF's intake and systemic inflammation.

### 2.5 Statistical Analysis

#### 2.5.1 Main Analyses

Each cytokine was assessed individually; outliers were identified and removed from the dataset to ensure accuracy, robustness, and reliability of the analyses. We defined outliers as those values that were greater than 1.5 times the interquartile range (IQR) above the upper quartile. Regarding cytokine measurements, only IL‐6 had values below the limit of detection, which were replaced with LOD divided by the square root of 2 (2.6/√2≈1.84), a single imputation method for left‐censored biomarker data. This approach is widely accepted when the proportion of censored values is low (e.g., < 25%) (Sigma‐Aldrich, n.d.; Whitcomb and Schisterman 2008; Hornung and Reed 1990). The other cytokines had no values below the LOD.

Generalized additive models (GAM) were developed to assess the linearity of the association between UPF intake and cytokine levels. Some of the associations appeared non‐linear (Figure S2), therefore we grouped participants into tertiles of UPF intake. The choice of the tertiles was based on the relatively small sample size of our study, preventing us from using a larger number of categories (e.g., quartiles or quintiles). Multiple generalized linear regression models were conducted using the tertiles of UPF intake as the main exposure and cytokine levels as the outcome (one at a time). Model 1 included only the age and sex of the child, and Model 2 included all the covariates detailed above.

#### 2.5.2 Stratified Analyses

We evaluated the interactions between UPF (continuous) and sex, age, and carer's education, using a likelihood ratio test; comparing models with and without a multiplicative interaction term (one at a time). We then conducted stratified analyses separately for girls and boys, by age (7 years, 8 years and 9–10 years), and by carer's education (< 12 years and ≥ 12 years).

#### 2.5.3 Sensitivity Analyses

In a set of sensitivity analyses, we further adjusted the models for BMI *Z*‐Score. Moreover, in another set of analyses, we restricted the analyses to those participants who were not exposed to environmental tobacco smoke.

All the statistical analyses were conducted with R Version (4.4.2).

## 3 Results

A total of 450 children were included in this study. The study population characteristics are described in Table 1. The majority of the children were 8 years old (52%); 45% were from an indigenous background, and 22.4% had a chronic disease diagnosis. The mean UPF intake was 29% (SD = 10.5%) of total energy intake. The children in the first tertile had an intake of 0%–24.5%, the second tertile from 24.6% to 33.7%, and the third tertile 33.8%–59.1%.

**TABLE 1:** Sociodemographic characteristics of the cohort.

UPF consumption	Overall (*n* = 450)	T1 (*n* = 150)	T2 (*n* = 150)	T3 (*n* = 150)	p*‐value* *^a^*
Age (years)					
7	146 (32%)	46 (31%)	48 (32%)	52 (35%)	0.3
8	233 (52%)	77 (51%)	78 (51%)	78 (52%)	
9	70 (15.8%)	28 (19%)	24 (16%)	18 (12%)	
10	1 (0.2%)	0 (0%)	1 (0.7%)	0 (0%)	
Girls	226 (50.2%)	71 (47%)	79 (53%)	76 (51%)	0.6
BMI kg/m^2^ (mean)	19.1	19.4	19.2	18.9	
BMI‐*Z* score (mean)	1.25	1.34	1.27	1.14	0.5
Mother's education (years)					
> 12	186 (41.4%)	70 (47%)	61 (41%)	55 (37%)	0.2
< 12	264 (58.6%)	80 (53%)	89 (59%)	95 (63%)	
Healthcare system					
Public	431 (95.7%)	144 (96%)	143 (95.3%)	144 (96%)	0.9
Private and armed forces	12 (2.6%)	6 (4%)	4 (2.7%)	2 (1.3%)	
Other	7 (1.5%)	0 (0%)	3 (2%)	4 (2.7%)	
Indigenous background	204 (45.3%)	72 (48%)	63 (42%)	69 (46%)	
Chronic disease diagnosis	101 (22.4%)	36 (24%)	36 (24%)	29 (19%)	0.5
UPF (% energy intake)		0%–24.5%	24.6%–33.7%	33.8%–59.1%	

Cytokines (pg/mL)	Median/IQR				
IL‐12 (*n* = 447)	15.7/6.41	15.7/6.28	15.1/6.37	16.2/6.38	0.12
IL‐10 (*n* = 438)	10.4/2.52	10.4/2.19	10.2/2.89	10.6/2.48	**0.01**
IL‐8 (*n* = 426)	20.7/7.55	20.8/6.75	19.9/8.20	21.1/7.08	0.32
IL‐6 (*n* = 432)	7.3/5.99	7.13/6.25	7.18/6.44	7.61/4.23	0.21
IL‐1β (*n* = 431)	13.2/5.83	13.3/5.57	12.9/5.83	14.9/5.92	**0.008**
TNF‐α (*n* = 445)	14.3/4.87	14.7/4.77	13.6/4.95	15.0/4.71	**0.02**

^a^ *p*‐value for ANOVA across tertiles of UPF. Median cytokine values. The *n* of each cytokine differs since they had a different number of outliers.

**FIGURE 1:**
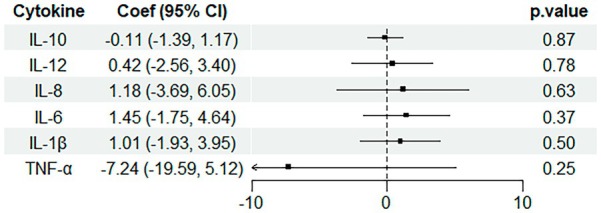
Linear associations between UPF intake (% energy intake, continuous) and six cytokine levels in children aged 7–9 years old (*n* = 450). Estimates from multiple linear regressions adjusted for sex, age, carer's education, healthcare affiliation, indigenous group, and chronic disease diagnosis.

**FIGURE 2:**
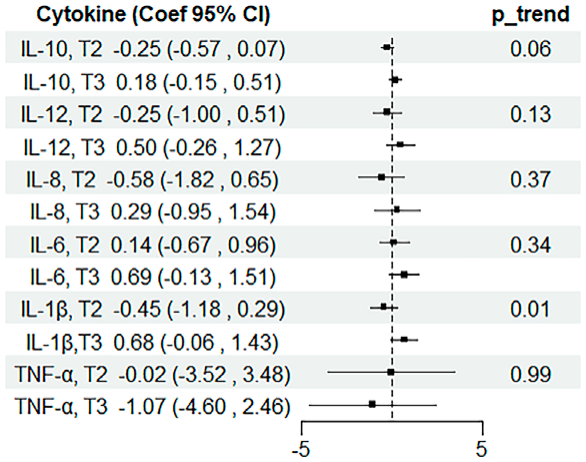
Associations between tertiles of UPF intake (% energy intake, reference = tertile 1) and six cytokine levels in children aged 7–9 years (*n* = 450). Estimates from multiple linear regressions adjusted for sex, age, carer's education, healthcare affiliation, indigenous group, and chronic disease diagnosis, using UPF as a categorical variable (tertiles of intake). Reference is the lowest tertile (T1).

There was no significant association between UPF intake and cytokine levels, neither when taking UPF as a continuous or categorical (tertile) variable in model 1 (Figure 1 and Table S2). In model 2, the overall differences across tertiles showed a suggestive trend for IL‐1β (Figure 2 and Table S2).

### 3.1 Stratified Analysis

**FIGURE 3:**
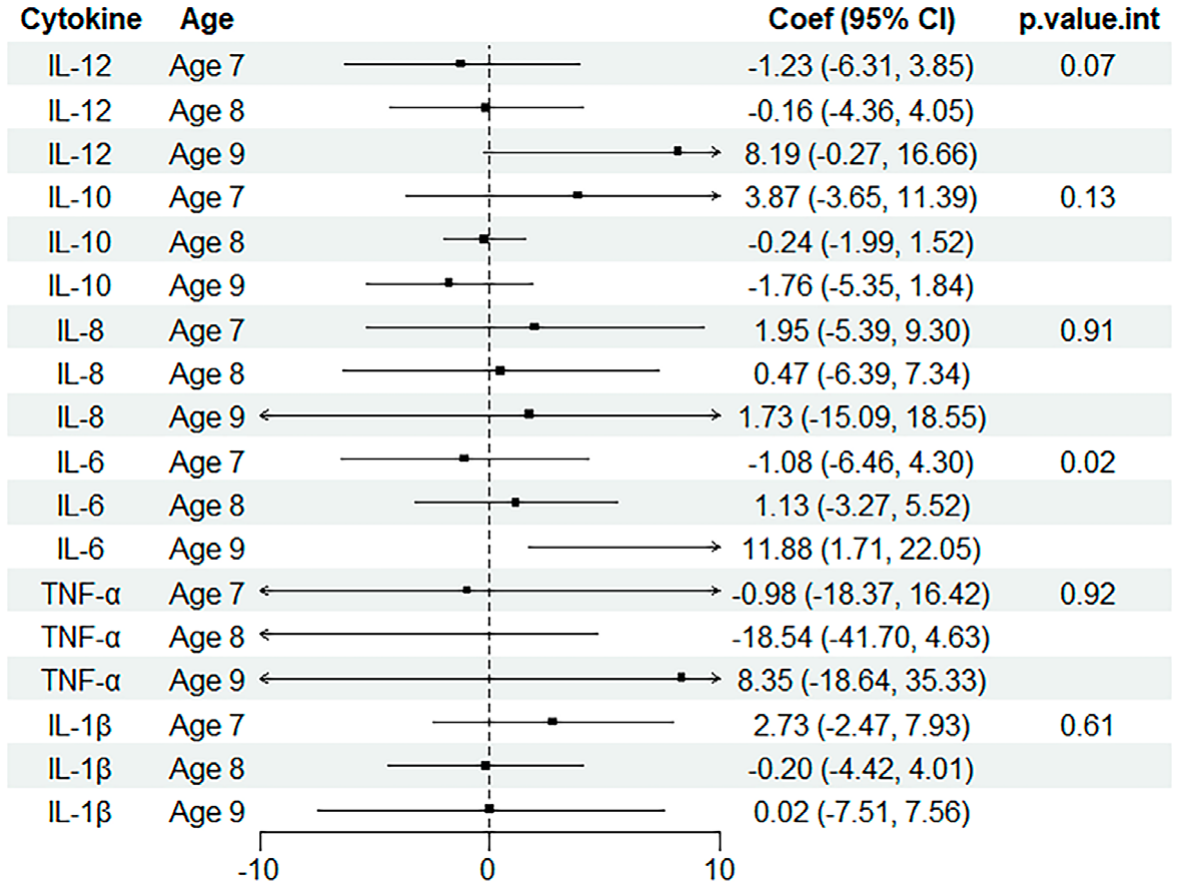
Cytokines estimates and ultra‐processed food intake stratified by age. ^†^Multiple linear regression including UPF as a continuous outcome, each specified cytokine as continuous exposure and Model 2 covariates: sex, age, carer's education, healthcare affiliation, indigenous group, chronic disease diagnosis. Stratification by ages 7, 8, and 9 (due to the very small number of children aged 10, they were grouped with the children aged 9 in the group “Age 9”).

We found a significant interaction between UPF and age (Figure 3) for IL‐6 and suggestive for IL‐12: an increase in the following cytokines was associated with UPF in the oldest age group only (≥ 9 years old; *n* = 71): IL‐12 (8.19, (0.27, 16.66) *p* = 0.04; *p*‐value interaction = 0.07) and IL‐6 (11.88, (1.71, 22.05) *p* = 0.01; *p*‐value interaction = 0.02). Despite the overall trend being significant, each association was not linear; when comparing T2 vs. T1 and T3 vs. T1, the associations were not significant.

There was no evidence of interaction with the sex of the child, or with the carer's education (Tables S3.1 and S3.2).

### 3.2 Sensitivity Analysis

Further adjustment of our analyses for BMI‐*z*‐score and restricting the analyses to children not exposed to environmental tobacco smoke did not change our findings notably (Tables S4.1 and S4.2).

## 4 Discussion

This study is one of the first to evaluate the relationship between UPF intake and inflammatory biomarkers in primary school‐aged children. Most results were non‐significant, only suggestive of a positive association between high UPF intake and IL‐1β and also for IL‐6 and IL‐12 in the older children only (≥ 9 years).

In our study population of school‐aged children in the city of Arica, Chile, we found that almost a third (29%) of their energy intake was coming from UPF. This is substantial, but overall lower than in other countries such as Brazil, which showed a share of 69.6% of the diet of children aged 7–9 years coming from UPF (Fonseca et al. 2023). Likewise, in the United Kingdom, 59.4% of the total energy intake came from UPF in a cohort of 7‐year‐old children (Conway et al. 2024), and in a Canadian cohort of 5‐year‐old children, the UPF share was 45% (Chen et al. 2025). On the other hand, our study reported higher UPF intake than in Italy, where it was reported that 23%–25% of the energy intake of children was coming from UPF in 5‐year old children (Ruggiero et al. 2021). To account for potential non‐linear associations between UPF intake and inflammatory biomarkers, UPF consumption was categorized into tertiles based on its distribution in the study population. This approach facilitated the identification of threshold effects and improved interpretability by allowing comparisons across low, medium, and high intake groups. While categorization may lead to some loss of statistical power and granularity compared to continuous modeling, it is a common method in nutritional epidemiology and remains valuable for identifying public health‐relevant patterns of dietary exposure (Santos Martins et al. 2020; Silva‐Luis et al. 2024). Systemic inflammation has been implicated in the pathogenesis of a number of non‐communicable diseases, highlighting potential concerns regarding inflammation in children. For instance, it has been shown that higher levels of IL‐1β in younger ages (4 to 17 years‐old) were associated with future risk of rheumatoid arthritis, inflammatory bowel disease, and osteopenia in adults (Pascual et al. 2005). TNF‐α has a complex role in cancer and has been shown to contribute to insulin resistance and lipid metabolism, both in adults and children (Popa et al. 2007). On the other hand, IL‐10 is widely known as an anti‐inflammatory cytokine and immunosuppressive cytokine since it promotes β cell differentiation, helping in tissue regeneration (Moudgil and Choubey 2011), and inhibits the production of proinflammatory cytokines such as IL‐1β, IL‐6, IL‐8, and TNF‐α (Hopkins 2003).

Nevertheless, IL‐10 has been described as a “context dependent” cytokine, since it may also act as an immunostimulatory cytokine in certain diseases such as cancer or Crohn's disease (Carlini et al. 2023). Additionally, IL‐10 plays a complex role in the immune system: it is recognized both as a powerful anti‐inflammatory and immunosuppressive cytokine, as well as having the potential to exhibit immunostimulatory properties. The sources of IL‐10, the target cells it influences, and the timing and location of its secretion are key factors that activate distinct signaling pathways, each contributing to either the inhibition or activation of immune cells. In our study, IL‐10 levels varied across tertiles of UPF intake, with the third tertile of UPF intake showing higher IL‐10 levels compared with the first tertile. IL‐10 has a key role in immune modulation and maintaining homeostasis, primarily exerting anti‐inflammatory effects through the JAK1/TYK2–STAT3 signaling pathway, which promotes anti‐inflammatory gene expression and limits excessive immune activation. The observed IL‐10 elevation likely represents a compensatory, homeostatic response to low‐grade inflammation potentially induced by higher UPF intake. This interpretation is supported by previous studies in children reporting similar patterns of increased IL‐10 in relation to UPF exposure (Carlini et al. 2023; Hutchins et al. 2013), suggesting a complex immunological environment involving both pro‐ and anti‐inflammatory signals. The role of IL‐10 in childhood in the etiology of future chronic disease remains to be elucidated.

An accumulating body of evidence shows associations between UPF intake and a range of adverse health outcomes in adolescents and, to a lesser extent, children. Most studies have focused on overall and central obesity (Cunha et al. 2018), blood pressure (Tandon et al. 2022), lipid (Tandon et al. 2022) and glucose metabolism (Tandon et al. 2022), and non‐alcoholic fatty liver disease (NAFLD) (Zhao et al. 2021), and were mainly conducted in adolescents aged 13 years or older. Most studies in adolescents or children have been conducted in Brazil. One article reported that UPF intake was associated with an increase in total cholesterol and LDL‐cholesterol in children from preschool to school age (Rauber et al. 2015). In contrast, another study, also in Brazil, did not find any association between UPF and LDL‐cholesterol (Gadelha et al. 2019). Adolescents with a high UPF intake (quintile 5 versus quintile 1) had significantly higher BMI and increased odds of being obese in another study (Louzada et al. 2015). Higher UPF consumption has also been associated with elevated blood glucose levels and BMI‐for‐age in adolescents (Cunha et al. 2018).

Only a few studies report associations with inflammatory biomarkers, particularly cytokines (Santos Martins et al. 2020; Silva‐Luis et al. 2024). A study in Brazil found that adolescents in the highest tertile of UPF intake had a 79% increase in IL‐8 levels when compared to those in the first tertile (Santos Martins et al. 2020). Another study conducted in 7–10‐yearyear‐old children has shown that IL‐17 and IL‐10 levels were higher in the second tertile of the UPF intake (Silva‐Luis et al. 2024).

## 5 Strengths and Limitations

This is one of the first studies evaluating the association of UPF intake with systemic inflammation in primary school children. Furthermore, this study was based on a well‐characterized cohort with data on a wide range of covariates. It is also the first to describe UPF intake in children and its relation with inflammatory biomarkers in Chile.

Our study also faced some limitations. Our cross‐sectional study design had limited capability to establish causality of the associations. Moreover, the relatively small sample size limited the statistical power, especially for the stratified analyses by age, where only 71 children were aged 9–10 years. Furthermore, the UPF intake was assessed through a FFQ, which can result in measurement errors due to recall bias, which is an inherent limitation of FFQs (Kipnis et al. 2003). This might be particularly true when responses are provided by caregivers on behalf of children, since they may have limited knowledge of meals eaten outside the home or in school settings. A frequent error is underreporting of certain foods—especially discretionary snacks or ultra‐processed products—due to memory lapses or “social desirability” (Rosi et al. 2022). As a result, the true intake of UPFs may be underestimated, and this potential misclassification should be considered when interpreting the dietary associations observed. An additional limitation of this FFQ is that it was validated in adults; however, this is the first study to use it in a population of children. Finally, given the relatively large proportion of loss to follow‐up, selection bias might be present, which can bias the estimates of the association between UPF and cytokines, and limits the generalizability of the findings, in particular the descriptive analyses.

## 6 Conclusion

Our findings indicated weak potential associations between higher UPF consumption and some cytokine levels in school‐aged children. These inflammatory markers are important for understanding potential long‐term health consequences, especially in terms of immune function and future risk of chronic disease. There is a need for more comprehensive research in pediatric populations to explore the impact of UPF on health outcomes and underlying mechanisms such as inflammation. Since global consumption of UPFs continues to rise, it is critical to prioritize dietary interventions that promote healthier, minimally processed food choices for children, aiming to reduce the long‐term risks of chronic diseases and support optimal growth and development. Further studies on the topic should prioritize longitudinal and larger cohort studies with enhanced statistical power to better establish causal relationships between UPF intake and inflammation. Additionally, employing more objective and accurate measures of UPF consumption, detailed food diaries validated by multiple sources, could reduce biases associated with dietary assessments. Such studies should also aim to minimize selection and recall biases to provide more robust and generalizable evidence on the long‐term health impacts of UPF intake in children.

## 7 Key Messages

1. These findings suggest systemic inflammation as a potential mechanism through which UPF consumption negatively affects children's health
2. A significant trend was found between UPF consumption and IL‐1β levels across increasing tertiles of intake, indicating a dose‐response relationship
3. Age may influence the inflammatory response to UPFs, with IL‐6 levels significantly associated with UPF intake among children aged 9 years and older.

## Author Contributions

**Camila Awad:** conceptualization (equal), data curation (lead), formal analysis (equal), investigation (lead), methodology (lead), validation (lead), visualization (lead), writing – original draft (equal), writing – review and editing (equal). **Paola Rubilar:** funding acquisition (lead), project administration (lead), resources (lead). **Macarena Hirmas‐Adauy:** project administration (equal). **Verónica Iglesias:** funding acquisition (equal), project administration (equal). **María Pía Muñoz:** investigation (supporting). **Mauricio A. Retamal:** methodology (equal). **Cristóbal Carvajal:** data curation (lead). **Payam Dadvand:** conceptualization (equal), formal analysis (equal), investigation (equal), supervision (equal), writing – original draft (equal), writing – review and editing (equal). **Camille Lassale:** conceptualization (equal), formal analysis (equal), investigation (equal), supervision (equal), writing – original draft (equal), writing – review and editing (equal).

## Ethics Statement

This study was approved by the Scientific Ethics Committee of the University of Desarrollo, Santiago, Chile (Approval number: 2022.81).

## Conflicts of Interest

The authors declare no conflicts of interest.

## Supporting information


**Data S1:** fsn370795‐sup‐0001‐Supinfo01.docx.

## Data Availability

Authors elect not to share data. Research data are not shared.
