# Supplementary material for: Ultra‐Processed Foods and Markers of Systemic Inflammation in Children
Source: Food Sci Nutr. 2025 Sep 1;13(9):e70795. doi: 10.1002/fsn3.70795 (PMC12401134; doi:10.1002/fsn3.70795)
Supplement: Supplementary file 1 — Data S1: fsn370795‐sup‐0001‐Supinfo01.docx. [file FSN3-13-e70795-s001.docx]

**Supplementary Material**

**"Ultra-Processed Foods and Markers of Systemic Inflammation in Children"**

**Supplementary Table 1: Food Frequency Questionnaire**

Next, we will ask about the frequency of food consumption that your child has had on average over the last 12 months. First, we will ask about the frequency of consumption of each food, categorizing them into four-time options: "never," "per month," "per week," or "per day." You should only select one option for each food. Then, we will ask how many times each food is consumed, based on the selected time option: If "never" is selected, you should mark the corresponding checkbox for the food and continue to the next one. If "per month" is selected, we will ask whether it is consumed "1 time or less" or "2 to 3 times." Only one checkbox should be marked with an X, and then you can continue with the next food. The same procedure applies for the options "per week" or "per day." Therefore, only one frequency option should be selected for each food; it is not possible to mark two options.

| **Consumption in the last**  **12 months** | Neve  r | Once a  mont  h | 2-3 times  a month | 1-2 times a week | 3-4 times a week | Onc e daily | 2-3 time s daily | 4-5 dail y | 6+ times a day |
| --- | --- | --- | --- | --- | --- | --- | --- | --- | --- |
| Whole milk |  |  |  |  |  |  |  |  |  |
| Semi/Skimme  d milk |  |  |  |  |  |  |  |  |  |
| Eggs (or preparations with them) |  |  |  |  |  |  |  |  |  |
| Red meat or pork low fat |  |  |  |  |  |  |  |  |  |
| Red meat or pork **high fat (sausages, burgers)** |  |  |  |  |  |  |  |  |  |
| Turkey |  |  |  |  |  |  |  |  |  |
| Chicken |  |  |  |  |  |  |  |  |  |
| White fish |  |  |  |  |  |  |  |  |  |
| Blue fish |  |  |  |  |  |  |  |  |  |
| Seafood |  |  |  |  |  |  |  |  |  |
| Vegetables general |  |  |  |  |  |  |  |  |  |
| Courgette |  |  |  |  |  |  |  |  |  |
| Tomato |  |  |  |  |  |  |  |  |  |
| Cucumber |  |  |  |  |  |  |  |  |  |
| Lettuce |  |  |  |  |  |  |  |  |  |
| Corn |  |  |  |  |  |  |  |  |  |
| Carrot |  |  |  |  |  |  |  |  |  |
| Beetroot |  |  |  |  |  |  |  |  |  |
| Fruits in general |  |  |  |  |  |  |  |  |  |
| Banana |  |  |  |  |  |  |  |  |  |
| Orange |  |  |  |  |  |  |  |  |  |
| Apple |  |  |  |  |  |  |  |  |  |
| Peach |  |  |  |  |  |  |  |  |  |
| Grape |  |  |  |  |  |  |  |  |  |
| Strawberry |  |  |  |  |  |  |  |  |  |
| Melon |  |  |  |  |  |  |  |  |  |
| Watermelon |  |  |  |  |  |  |  |  |  |
| Pear |  |  |  |  |  |  |  |  |  |
| Mango |  |  |  |  |  |  |  |  |  |
| Prickly pear |  |  |  |  |  |  |  |  |  |
| Rice |  |  |  |  |  |  |  |  |  |
| Dried fruit |  |  |  |  |  |  |  |  |  |
| Legumes |  |  |  |  |  |  |  |  |  |
| Oils (olive, sunflower) |  |  |  |  |  |  |  |  |  |
| Other fats **(margarine**, butter) |  |  |  |  |  |  |  |  |  |
| Pasta |  |  |  |  |  |  |  |  |  |
| **Cereals** |  |  |  |  |  |  |  |  |  |
| **Pastries** |  |  |  |  |  |  |  |  |  |
| **Sugar** |  |  |  |  |  |  |  |  |  |
| **Cookies or baked goods** |  |  |  |  |  |  |  |  |  |
| **Sugary drinks** |  |  |  |  |  |  |  |  |  |
| Water |  |  |  |  |  |  |  |  |  |

We conducted this 44-item FFQ, where we classified seven as UPF (NOVA group 4): high fat red meat (hamburgers and sausages), margarine, cereals, pastries, sugar, cooked or baked goods, sugary drinks. All the food groups were calculated from portions of intake, according to the Chilean Food Composition Table, where macro and micronutrients, are available per portion size. Kilocalories from each food item were calculated accordingly. Then these seven food groups were grouped as UPF. The total daily energy intake from UPF items was added and divided by the total estimated energy intake from all FFQ items, resulting in the percentage of energy from UPFs (%kcal) for each participant. To convert frequency options into daily portions, we multiplied by the appropriate fraction (number of times per day/week/month divided by number of days in week/month when appropriate). When the possible answer was a range (e.g. 1-2 times a week) the midpoint value was chosen (1.5 in this example) and divided by number of days in the week (7) or month (30.5), accordingly.


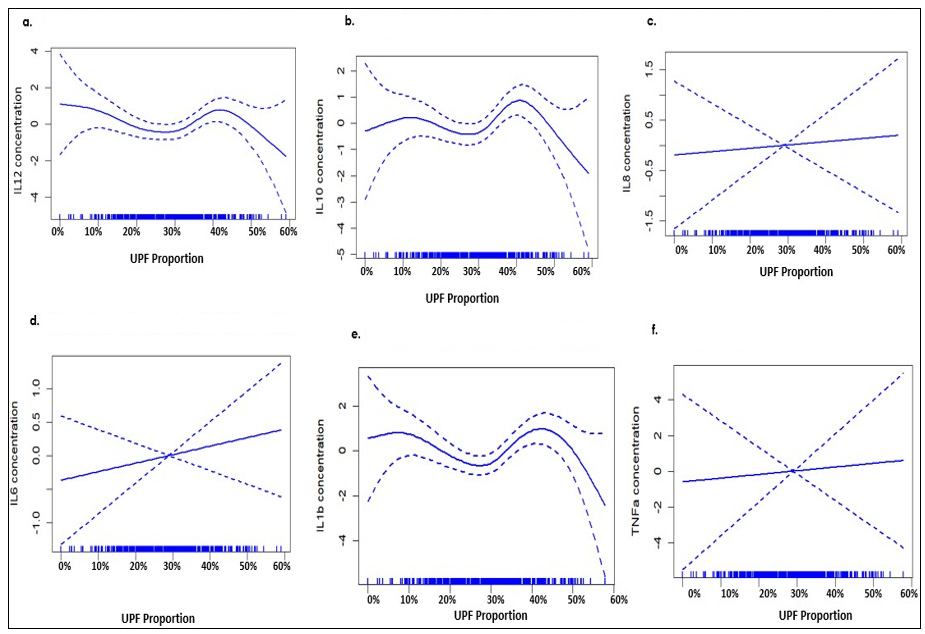
**Supplementary Figure 1**: Generalized Additive Models of the associations between UPF intake and 6 cytokine levels in children ^†^

^†^Covariates included in the model: age, mother/guardian’s education, healthcare affiliation, indigenous group, chronic disease diagnosis. The outcome cytokine is: a. IL-12; b. IL-10; c. IL-8 ; d. IL-6, e. IL-1β ; f. TNF-α

**Supplementary Table 2:** Associations from linear regression models between Ultra Processed Foods (tertiles and continuous) and cytokines levels

| **Cytokine/**  **Tertiles UPF Intake (% energy intake)** | **Tertile 1** | **Tertile 2** | **Tertile 3** | **p-value trend ^§^** | **Continuous UPF (% energy**  **intake)** | **Cont. pvalue ^¶^** |
| --- | --- | --- | --- | --- | --- | --- |
|  | **<24.5%** | **24.6%-**  **33.7%** | **33.8%-**  **59.1%** |  |  |  |
| **IL-12** |  |  |  |  |  |  |
| Model 1 †  *β (95% CI)* | Reference | -0.23 (-0.98,  0.52) | 0.50 (-0.22,  1.28) | 0.17 | 0.55 (-2.38,  3.49) | 0.71 |
| Model 2 ‡  *β (95% CI)* | Reference | -0.25  (-1.00,  0.51) | 0.50 (-0.26,  1.27) | 0.13 | 0.42  (-2.56, 3,4) | 0.78 |
| **IL-10** |  |  |  |  |  |  |
| Model 1 β *(95% CI)* | Reference | -0.27 (-0.94, 0.4) | 0.51 (-0.16,  1.19) | 0.14 | 0.99 (-1.65,  3.64) | 0.46 |
| Model 2  *β (95% CI)* | Reference | -0.25  (-0.57,  0.07) | 0.18  (-0.15,  0.51) | 0.06 | -0.11 (-1.39,  1.17) | 0.87 |
| **IL-8** |  |  |  |  |  |  |
| Model 1  *β (95% CI)* | Reference | -0.60 (-1.83,  0.62) | 0.17 (-1.06,  1.40) | 0.79 | 0.58 (-4.23,  5.39) | 0.81 |
| Model 2  *β (95% CI)* | Reference | -0.58 (-1.82,  0.65) | 0.29 (-0.95,  1.54) | 0.37 | 1.18 (-3.69,  6.05) | 0.63 |
| **IL-6** |  |  |  |  |  |  |
| Model 1  *β (95% CI)* | Reference | 0.14 (-0.68,  0.94) | 0.68 (-0.12,  1.49) | 0.09 | 1.43 (-1.55,  4.42) | 0.34 |
| Model 2 *β (95% CI)* | Reference | 0.14  (-0.67,  0.96) | 0.69  (-0.13,  1.51) | 0.34 | 1.45 (-1.75,  4.64) | 0.37 |
| **IL1-β** |  |  |  |  |  |  |
| Model 1 *β (95% CI)* | Reference | -0.45 (-1.18,  0.28) | 0.65 (-0.08,  1.38) | 0.08 | 1.11 (-  1.77,4.01) | 0.44 |
| Model 2  *β (95% CI)* | Reference | -0.45 (-1.18,  0.29) | 0.68 (-0.06,  1.43) | **0.01** | 1.01 (-1.93,  3.95) | 0.50 |
| **TNF-α** |  |  |  |  |  |  |
| Model 1  *β (95% CI)* | Reference | 0.13 (-3.98,  4.24) | 0.57 (-3.54,  4.69) | 0.79 | -7.01 (-19.2,  5.15) | 0.25 |
| Model 2 β *(95% CI)* | Reference | -0.02 (-3.52,  3.48) | -1.07 (-4.60,  2.46) | 0.99 | -7.24 (-  19.59, 5.12) | 0.25 |

†Model 1 includes the following covariates: sex, age

‡ Model 2 includes the following covariates: sex, age, mother/guardian’s education, healthcare system, indigenous group, chronic disease diagnosis.

^§^p-value for trend was calculated using linear regression, with UPF % of energy intake, categorized into tertiles (1, 2, and 3), using tertile 1 as a reference value. ^¶^p-value was calculated using linear regression, with UPF % energy intake as a continuous variable.

**Supplementary Tables 3:** Interaction test ^†^

Table 3.1: Interaction term by sex

| **Cytokine** | ***p-value*** |
| --- | --- |
| **IL-12** | 0.36 |
| **IL-10** | 0.34 |
| **IL-8** | 0.46 |
| **IL-6** | 0.56 |
| **IL1-β** | 0.34 |
| **TNF-α** | 0.96 |

Table 3.2: Interaction term by carer’s education

| **Cytokine** | ***p-value*** |
| --- | --- |
| **IL-12** | 0.60 |
| **IL-10** | 0.71 |
| **IL-8** | 0.91 |
| **IL-6** | 0.76 |
| **IL-1β** | 0.91 |
| **TNF-α** | 0.89 |

Table 3.3 Interaction term by age

| **Cytokine** | ***p-value*** |
| --- | --- |
| **IL-12** | 0.07 |
| **IL-10** | 0.13 |
| **IL-8** | 0.91 |
| **IL-6** | 0.02 |
| **IL1-β** | 0.61 |
| **TNF-α** | 0.92 |

^†^Multiple linear regression of Model 2: adjusted by sex, age, carer’s education, healthcare affiliation, indigenous group, chronic disease diagnosis, stratifying by sex, carer’s education and age.

**Supplementary Tables 4**: Sensitivity Analyses of Model 2 (continuous exposure of UPF) further including or excluding specified covariates ^†^

Table 4.1: Including BMI z-score

| **Cytokine** | ***β (95% CI)*** | ***p-value*** |
| --- | --- | --- |
| **IL-12** | 0.30 (-2.7 3.3) | 0.84 |
| **IL-10** | 0.93 (-1.78, 3.64) | 0.50 |
| **IL-8** | 1.27 (-3.69, 6.16) | 0.62 |
| **IL-6** | 1.16 (-1.76, 4.60) | 0.47 |
| **IL1-β** | 0.93 (-2.02, 3.90) | 0.53 |
| **TNF-α** | -6.85 (-15.45, 17.70) | 0.96 |

Table 4.2: Including Exposure to Tobacco Smoke the last 72 hours

| **Cytokine** | ***β (95% CI)*** | ***p-value*** |
| --- | --- | --- |
| **IL-12** | 0.37 (-2.64 3.38) | 0.81 |
| **IL-10** | -0.11 (-1.40, 1.19) | 0.87 |
| **IL-8** | 1.28 (-3.66, 6.21) | 0.61 |
| **IL-6** | 1.17 (-2.06, 4.39) | 0.48 |
| **IL1-β** | 0.82 (-2.15, 3.79) | 0.59 |
| **TNF-α** | -8.25 (-22.4, 5.80) | 0.25 |

Table 4.3: Excluding Chronic Disease Diagnosis

| **Cytokine** | ***β (95% CI)*** | ***p-value*** |
| --- | --- | --- |
| **IL-12** | 0.51 (-2.44 3.47) | 0.73 |
| **IL-10** | 0.98 (-1.69, 3.66) | 0.47 |
| **IL-8** | 0.82 (-4.01, 5.67) | 0.73 |
| **IL-6** | 1.42 (-1.75, 4.59) | 0.37 |
| **IL1-β** | 1.16 (-1.76, 4.08) | 0.43 |
| **TNF-α** | -7.39 (-15.6, 17.02) | 0.29 |

Table 4.4: Excluding Carer’s Education

| **Cytokine** | ***β (95% CI)*** | ***p-value*** |
| --- | --- | --- |
| **IL-12** | 0.35 (-2.55, 3.34) | 0.81 |
| **IL-10** | -0.15 (-1.38, 1.17) | 0.82 |
| **IL-8** | 0.86 (-3.68, 6.04) | 0.73 |
| **IL-6** | 1.44 (-1.74, 4.63) | 0.37 |
| **IL1-β** | 0.97 (-1.92, 3.94) | 0.52 |
| **TNF-α** | -7.62 (-21.49, 6.26) | 0.28 |

Table 4.5: Excluding Ethnicity

| **Cytokine** | ***β (95% CI)*** | ***p-value*** |
| --- | --- | --- |
| **IL-12** | 0.42 (-2.55, 3.39) | 0.78 |
| **IL-10** | -0.10 (-1.84, 3.53) | 0.87 |
| **IL-8** | 1.18 (-3.68, 6.04) | 0.63 |
| **IL-6** | 1.44 (-1.74, 4.63) | 0.37 |
| **IL1-β** | 1.00 (-1.92, 3.94) | 0.50 |
| **TNF-α** | 0.26 (-16.17, 16.70) | 0.97 |

Table 4.6: Excluding Healthcare Affiliation

| **Cytokine** | ***β (95% CI)*** | ***p-value*** |
| --- | --- | --- |
| **IL-12** | 0.50 (-2.46, 3.47) | 0.74 |
| **IL-10** | 0.11 (-1.38, 1.67) | 0.87 |
| **IL-8** | 1.19 (-3.65, 6.04) | 0.63 |
| **IL-6** | 1.46 (-1.7^^[[1]](#footnote-1)^^, 4.65) | 0.37 |
| **IL1-β** | 1.00 (-1.91, 3.92) | 0.49 |
| **TNF-α** | -7.25 (-21.08, 6.57) | 0.30 |

1. [↑](#footnote-ref-1)
